# Psychopathic Traits and Sensation Seeking in Young General Population

**DOI:** 10.3390/bs16040504

**Published:** 2026-03-27

**Authors:** María de la Villa Moral-Jiménez, Ana Sierra Sánchez

**Affiliations:** Department of Psychology, University of Oviedo, 33003 Oviedo, Spain; uo271075@uniovi.es

**Keywords:** psychopathy, sensation seeking, disinhibition, subclinical population

## Abstract

Background: The construct of psychopathy encompasses the manifestation of psychopathic traits in subclinical populations related to sensation seeking. This study aims to examine the relationship between these two constructs and to determine whether differences exist as a function of age and gender. Method: A total of 345 participants aged between 18 and 40 years (M = 24.72, SD = 5.60) were recruited and completed the Levenson Self-Report Psychopathy Scale (LSRP) and the Brief Sensation Seeking Scale (BSS). Results: Positive and significant correlations were found between the three psychopathy factors and the disinhibition subscale. Specifically, the interpersonal and behavioural factors were positively associated with disinhibition, whereas the affective factor showed a negative association. Gender differences were observed, with men scoring higher on the behavioural factor of psychopathy, disinhibition and sensation seeking. Conclusions: The findings confirm the relationship between psychopathy and sensation seeking in a subclinical population and contribute to a better understanding of their potential implications.

## 1. Introduction

Psychopathy is an extremely complex personality construct that encompasses aspects beyond criminality and is recognised as a clinical entity with multiple differential nuances. Despite extensive research on the subject, there are still no unified conceptual and terminological criteria (Fernández & Calderón, 2024; Gatner et al., 2016; B. Pérez et al., 2016; Weidacker et al., 2017). Since Pinel’s well-known conceptualisation of psychopathy as mania without delusions, referring to an alteration of affective functions without impairment of executive functions (memory, perception, and intelligence) (S. López, 2013; Patrick, 2018), numerous labels have emerged (B. Pérez, 2014; Torrubia & Cuquerella, 2008). A turning point in the conception of psychopathy came with Cleckley (1976), who was the first author to distinguish between criminal and functional psychopathy and to emphasise affective and interpersonal characteristics, although he did not consider the presence of antisocial behaviour to be necessary (Cleckley, 1976; J. M. A. Rodríguez et al., 2018).

Different nosological entities with similar clinical characteristics coexist, such that three different meanings can be identified for what is commonly understood as psychopathy (S. López, 2013). The International Classification of Diseases uses the term Dissocial Personality Disorder (ICD-11) (World Health Organization, 2019), and the fifth revised edition of the Diagnostic and Statistical Manual of Mental Disorders (DSM-5-TR) of the American Psychological Association (American Psychological Association, 2022) refers to Antisocial Personality Disorder (ASPD) (De Brito et al., 2021). Finally, Robert D. Hare’s criteria for psychopathy are widely used. These are derived from Cleckley’s description of this personality disorder and served as the basis for the construction of the most widely used psychopathy assessment instrument to date—the Psychopathy Checklist (PCL)—and its revised version, the PCL-R (Hare, 1991; Mas & Vilela, 2022; B. Pérez, 2014; Reglero, 2020).

Within this theoretical framework, psychopathy is fundamentally understood as a personality disorder marked by emotional disturbance and lack of empathy, leading to serious difficulties in establishing emotional bonds with others. It is considered an early-onset pathology, stable over time, characterised by a pattern of dysfunctional behaviour that can affect different areas of an individual’s life (De Brito et al., 2021; J. M. A. Rodríguez et al., 2018). To understand the broad spectrum of traits that make up this construct, several defining characteristics are worth mentioning: (a) hostility towards the environment, which can lead to violent, cruel, and manipulative acts; (b) emotional insensitivity, lack of empathy, and absence of guilt; and (c) narcissism, impulsivity, and thrill seeking, which may lead to the transgression of social norms (Patrick, 2018). However, these core characteristics have been grouped according to the domains affected. As is well known, Hare et al. (1990) defined twenty traits in the PCL, dividing them into two factors. The first refers to the affective and interpersonal component and includes traits such as lack of remorse and inability to maintain affective relationships. At the interpersonal level, egocentricity, grandiosity, manipulation, and superficial charm stand out. The second factor comprises characteristics related to behavioural and lifestyle components, including impulsivity, need for stimulation, irresponsibility, and antisocial behaviour (B. Pérez, 2014; B. Pérez et al., 2022; Silva, 2009; Veal et al., 2021).

Based on this two-factor model, other explanatory models have been developed that further subdivide these components. In the case of the first factor, a distinction is made between affective and interpersonal dimensions, and some authors also propose a third, cognitive dimension focusing on narcissistic personality traits (R. Rodríguez & González-Trijueque, 2014; J. M. A. Rodríguez et al., 2018). Regarding the second factor, authors such as Cooke and Michie (2001) distinguish lifestyle traits (e.g., impulsivity and irresponsibility) from antisocial behaviour, even excluding the latter from the core defining characteristics of psychopathy by conceptualising it as a consequence rather than an essential component (Sellbom & Drislane, 2021).

On the other hand, the Levenson Self-Report Psychopathy Scale Scale for Psychopathy (LSRP) is widely used to assess psychopathic traits in non-institutionalised samples, such as those in this study; a distinctive feature is that its items contain no explicit reference to overt antisocial behaviour. It is applicable to nonclinical samples, with the likely limitation of the range associated with relatively low levels of psychopathic traits in community samples. According to Pozueco et al. (2013a, 2013b), clinical psychopathy has traditionally been linked to serious antisocial and criminal behaviours, whereas subclinical or integrated psychopathy exhibits the same personality traits (superficial charm, manipulation, and emotional coldness, among others) but adapted to society without the commission of serious crimes. Thus, the differences between subclinical psychopathy and criminal psychopathy are evident, primarily, in their behavioural aspects (Pozueco et al., 2013a). Thus, from a subclinical perspective, psychopathy represents a general personality trait in the general or civilian population (Benning et al., 2005), hence the suggestion that information on psychopathy can also be obtained from community samples (Malterer et al., 2010). In turn, psychopathic traits represent isolated characteristics—such as boldness and a lack of empathy—that are present in the general population without constituting a disorder or a personality structure. Specifically, the LSRP can measure the continuum between clinical, subclinical, and integrated psychopathy and psychopathic traits based on the transition from psychopathy as a severe clinical disorder toward a model of personality traits present in the general population. Unlike other clinical scales, the LSRP assesses primary psychopathy (tendency toward manipulation, selfishness, lack of empathy, and emotional coldness) and secondary psychopathy (impulsive lifestyle, irresponsibility, and low frustration tolerance) by considering this explanatory continuum.

Despite numerous studies, the causes of these traits remain unclear. The main hypotheses refer to biological predispositions and social factors. Research on genetic influences is inconclusive, as no direct causal relationship with the development of psychopathy has been firmly established (Veloso, 2021). Concerning neurobiological alterations, correlations have been found between psychopathic behaviours and structural brain abnormalities. Specifically, reductions in grey matter have been observed in the prefrontal cortex (related to impulse control) and in areas such as the amygdala and hippocampus (involved in emotional regulation and social behaviour). Altered physiological responses to stimulation have also been detected, including electrodermal activity, blink responses, and facial expressions (Calderón et al., 2019; Garofalo et al., 2021). Environmental and socialisation processes have also been identified as relevant factors in the development of psychopathy. In particular, the quality of attachment bonds, parenting style, and family and social environments are highlighted. Exposure to trauma at different developmental stages has been associated with the manifestation of clinical psychopathy (Whyte & Rehman, 2021). Emotional deprivation, absence of parental figures, and experiences of abuse or violence are among the factors that may predispose individuals to the development of psychopathic traits (De Brito et al., 2021). Reflecting the complexity of the construct of psychopathy, different types can be observed, with psychopaths being classified as primary or secondary (Luján et al., 2023; S. López, 2013; Sellbom & Drislane, 2021), successful or unsuccessful, criminal or adapted, etc. (S. López, 2013). Regarding the classification that distinguishes between criminal psychopathy and adapted or subclinical psychopathy, the distinction between the two concepts rests on whether or not they exhibit criminal, violent or delinquent behaviour, despite both having the usual emotional and interpersonal characteristics of a psychopath (M. J. López & Núñez, 2024). The subclinical psychopath is defined by their ability to manipulate those around them and by possessing the social skills necessary for proper social adaptation. They can be defined by their superficial charm, glibness, self-confidence, coldness in and control of situations and, above all, lack of empathy, although it is true that they may resort to violence if manipulation proves ineffective (see Benning et al., 2018; Miguez, 2014; Pozueco et al., 2013b). Authors such as Blötner et al. (2025) refer to focal correlates of psychopathy related to sadism. In turn, Araújo et al. (2025) estimate that highly psychopathic traits are related to antisocial behaviour, although this association is mediated by maladaptive beliefs.

Regarding the prevalence rate of subclinical psychopathy in the general Spanish population, Sanz et al. (2022) estimated it at approximately 1.65%. Román (2022) suggests that it may be an underdiagnosed condition, as social and legal factors converge and research outside prison settings presents methodological challenges. Furthermore, psychopathy is considered an egosyntonic condition, and much of the research has been conducted in non-institutionalised university samples, limiting comprehensive understanding (B. Pérez et al., 2016). According to Hare (2013), subclinical psychopathy is characterised by a high tendency towards sensation-seeking behaviour, as well as low empathy and low anxiety (Alamán & Pérez, 2021). Sensation seeking is a personality trait referring to the need for novel and intense stimulation and a tendency toward boredom, associated with the desire to feel alive and escape routine or monotonous tasks. In the context of psychopathy, it is also linked to recklessness, lack of planning, irresponsibility, and failure to fulfil family, work, and social obligations (Rolls et al., 2022; Silva, 2009). Although sensation seeking has been associated with antisocial and criminal behaviour, Cooke and Michie (2001) excluded it from the core defining criteria of psychopathy. Nevertheless, recent research supports a relationship between the two constructs. One of the earliest studies demonstrating this association was conducted by Haapasalo (1990), and more recently, Weidacker et al. (2017) reported that psychopathy was linked to multiple forms of impulsivity. This relationship appears particularly salient in young people, partly due to the transitional nature of adolescence—a developmental stage during which cognitive functions mature and the ability to consider long-term consequences develops, gradually promoting more prosocial behaviour (Bentacourt & Campos, 2015). Research on sensation seeking and risk-taking in young adults (aged 18–25) has yielded significant findings, although further investigation is still needed (Babad et al., 2021). While irresponsible and risky behaviours have also been observed in older adults, research in this area remains inconclusive (Silva, 2009). Finally, sensation-seeking behaviours associated with the two main psychopathy factors do not appear uniformly across men and women (Anderson et al., 2021). A lower prevalence of psychopathy has been reported in women, and although similar personality traits may be present, behavioural differences have been observed, with women generally displaying lower impulsivity (Reglero, 2020).

Taking previous research into account, the main objective of this study is to examine the relationship between subclinical psychopathy and sensation-seeking behaviours in a non-forensic population sample.

The specific objectives are: (1) to identify which factors of subclinical psychopathy show the strongest correlations with sensation seeking, considering its subscales (emotion seeking, excitement seeking, disinhibition, and boredom susceptibility); (2) to analyse differences in sensation seeking and psychopathic traits according to age; and (3) to examine potential gender differences in both psychopathic traits and sensation seeking.

The following research hypotheses are proposed: H1: A positive relationship is expected between sensation seeking and both the affective–interpersonal and behavioural components of psychopathy. H2: Younger participants are expected to score higher on both constructs. H3: Gender differences are anticipated, with men scoring higher than women on psychopathic traits and sensation seeking.

## 2. Materials and Methods

### 2.1. Design

An exploratory, descriptive, cross-sectional, ex post facto design was applied. A multivariate correlational design was used.

### 2.2. Participants

A total of 345 individuals selected through purposive sampling participated in the study, of whom 207 were women (60%) and 138 were men (40%), aged between 18 and 40 years (X = 24.72, SD = 5.6), and were distributed into different age groups according to the criteria of the American Academy of Pediatrics (see Sawyer et al., 2018), which refers to late adolescence as the ages 18 to 24 (n = 210, 60.86%, X = 20.99, SD = 1.6) and young adulthood as from 25 to 40 years (n = 135, 39.1%, X = 30.51, SD = 4.57) (American Academy of Pediatrics, 2019). With regard to other characteristics, only 5% (n = 18) of respondents were unemployed, while the rest were ‘studying and/or working’. Most participants had completed secondary school or intermediate vocational training (47.2%, n = 163) and university or advanced vocational training (36.8%, n = 127). In terms of marital status, almost half were in a relationship (46.9%, n = 162) or single (44.3%, n = 153). The distinction between clinical and subclinical psychopathy is based primarily on the intensity of traits, the individual’s functionality, and the presence of serious criminal or antisocial behaviour. This study analysed psychopathic traits or integrated psychopathy. To this end, in order to ensure that the sample is subclinical and to limit the inclusion of clinical profiles, we have applied measures to guarantee a subclinical sample. We have used community and university samples, not samples from prisons or forensic institutions, and a selection has been made based on scores, with individuals who have high scores on traits but do not reach the clinical diagnosis. Furthermore, we have taken into account exclusion criteria for characteristics associated with clinical outcomes such as criminal records or convictions for violent acts, since subclinical psychopathy is characterised by the avoidance of such direct criminality.

### 2.3. Measures

First, the Levenson Self-Report Psychopathy Scale (LSRP) was applied, which was specially designed to measure psychopathic personality traits in the general population, i.e., in non-institutionalised samples (Levenson et al., 1995). This measurement instrument was selected over others (PPI-S, SRP, etc.) due to its favourable psychometric properties, as it has good reliability values (α = 0.83 on the overall scale) and can be applied to a wide range of population samples (Camacho et al., 2011; Hauck-Filho & Teixeira, 2014). It consists of 26 items measured using a Likert scale, with 1 being ‘strongly disagree’ and 4 being ‘strongly agree.’ These items provide a total psychopathy score and are divided into two factors based on Hare’s Psychopathy Checklist (PCL). The first of these (16 items) corresponds to the affective–cognitive component, and the second (10 items) refers to the behavioural component, with Cronbach’s alpha reliability indices of 0.82 and 0.61, respectively (Camacho et al., 2011). In the case of the present study, and as a result of confirmatory factor analysis (CFA), which will be detailed below, three factors were obtained. The first showed a McDonald’s omega reliability index of 0.74, while the second factor obtained a reliability of 0.68 and the third a reliability of 0.64.

It is worth noting that the LSRP conceptualises psychopathy as linked to antagonistic tendencies and egocentrism; thus, in the study by Garofalo et al. (2019), significant associations were found with traits characteristic of narcissistic personality disorder, low self-control and identity integration, and problems in relational functioning. Regarding the factor structure, in the aforementioned study by Garofalo et al. (2019), confirmatory factor analysis corroborated the superiority of the LSRP’s three-factor model: egocentrism, insensitivity, and antisociality. On the other hand, the sensation-seeking variable was assessed using the Sensation Seeking Scale (Form V), adapted to the Spanish population in the study by J. Pérez and Torrubia (1986). This scale was a reworking by Zuckerman et al. (1978) of the original instrument for measuring this personality trait. It consists of 40 items measured by dichotomous ‘yes or no’ responses, providing an overall score for this variable with a reliability index of 0.91 (Zuckerman, 2007). According to the factorial structure, it consists of four subscales: *thrill seeking* (BEM) (α = 0.91), which measures the need for excitement and adventure; *excitement seeking* (BEX) (α = 0.80), which refers to daring experiences; *disinhibition* (DES) (α = 0.84), which relates to lack of restraint and spontaneity (especially in sexual matters); and, finally, *susceptibility* to boredom (SAB) (α = 0.74). The latter assesses the rejection of routine, i.e., the repetition of experiences (Gray & Wilson, 2007; J. Pérez & Torrubia, 1986; Zuckerman, 2007). In this study, after performing an exploratory factor analysis, a reliability of 0.81 was obtained for the emotion seeking (BEM) subscale, 0.48 for the excitement seeking (BEX) subscale, 0.69 for disinhibition (DES), and 0.55 for susceptibility to boredom (SAB), so that the BEX and SAB subscales were not used in the analyses.

### 2.4. Procedure

The strategy followed for data collection was primarily online, as all information was gathered using a questionnaire created on the Google Forms platform and disseminated through social media and personal contacts. To preserve data confidentiality, data was collected anonymously and used solely for research purposes. This study complies with ethical standards for research involving humans, in accordance with the ethical standards of the American Psychological Association (American Psychological Association, 2010) manual. Prior to completing the form, the informed consent clause was completed. Additionally, the explicit consent of the respondents was required to collect the data, checking a box to communicate their agreement, in accordance with Organic Law 3/2018 (Organic Law 3/2018, 2018).

### 2.5. Data Analysis

Once the data collection was complete, it was analysed in several phases. First, a confirmatory factor analysis of the instruments used in this research was performed using the JASP statistical programme. Firstly, the Levenson Self-Report Psychopathy Scale (LSRP) was studied, noting the absence of correction scales for scores in the Spanish population. For this reason, studies that have verified its validity in this type of population (see J. M. A. Rodríguez et al., 2018) were used to determine its factorial structure, taking the calculated factor weights as a reference. Furthermore, both in this psychometric review and in other studies (see Brinkley et al., 2001; Camacho et al., 2011), it has been observed that a third factor could be found in this scale, which would bring together those items that were not highly saturated in the first factor. Thus, the existence of three factors could be considered: an interpersonal component, behavioural or impulsivity component, and affective component. Taking this into account, and referring to J. M. A. Rodríguez et al. (2018), items 2, 3, 9, and 20 were excluded from the AFC because, as indicated in the reference study, they do not meet the saturation criteria for any of the factors. Subsequently, as a result of the present AFC, item 13 was also eliminated.

Secondly, a CFA of the Sensation Seeking Scale (Form V) was performed, placing each of the 40 items in its corresponding subscale to check the fit of these parameters. The reliability of the scale, whose values have been presented above, was also studied. The confirmatory factor analysis (CFA) was conducted using AMOS in SPSS version 30, with the Maximum Likelihood (ML) estimation method. Next, in order to determine the type of statistics (parametric or non-parametric) that would be needed to test the proposed hypotheses, the distribution of the sample population was checked using the Kolmogorov–Smirnov descriptive statistic. Finally, taking into account all the previous analyses, the hypotheses were tested. Prior to conducting correlational analyses, the distributions of the total subscale scores were examined for normality. Given the observed distributions and the characteristics of the data, Spearman’s rho was used to assess correlations between variables. In the first and third hypotheses, Spearman’s non-parametric Rho dependency measure was used to obtain the correlations between the different psychological variables. Although the distributions of the study variables were examined for normality, the non-parametric Mann–Whitney U test was used for group comparisons to ensure robustness given the characteristics of the data.

## 3. Results

### 3.1. Confirmatory Factor Analysis of the Instruments

In order to compare the models underlying the measurement instruments used, confirmatory factor analysis was performed on each of them. Thus, as shown below, the data structure effectively fits the factors proposed in both scales.

In the case of the Levenson Self-Report Psychopathy Scale (LSRP), the data fit the proposed factorial structure well, thus confirming the study by J. M. A. Rodríguez et al. (2018), which was taken as a reference and which considered the existence of three factors instead of the two proposed by Levenson et al. (1995). Specifically, robust results were obtained that confirmed the fit of the proposed structural factor model to the correlations between the variables in the dataset. However, in the Lambdas of each item in its corresponding factor, it was observed that, although all of them were significant, the one corresponding to item 13 had a negative sign. For this reason, and considering that this would pose a problem in the use of the scale, it was removed.

The confirmatory factor analysis was repeated, and once again, the chi-square (χ^2^) fit index values between the hypothesised model and the available data were found to be statistically significant (*p* < 0.001), with a high χ^2^ value (χ^2^ = 337.26) and 186 degrees of freedom (df). Therefore, the sample variables are sufficiently correlated with each other to perform factor analysis (<χ^2^/df). On the other hand, the descriptive goodness-of-fit indices confirm the adequacy of the factorial model for the data, finding robust results for the Comparative Fit Index (CFI), which should be greater than 0.90 (CFI = 0.91), and for the RMSEA, which is effectively less than 0.05 (RMSEA = 0.048) (see Table 1).

With regard to the Sensation Seeking Scale (BSS), the results of the confirmatory factor analysis also show a good fit of the data to the factorial structure underlying the model, thus confirming the existence of four factors, as shown in Table 2. In this case, the χ^2^ fit index is statistically significant (*p* < 0.001), with a χ^2^ value of 1031.46 and 734 degrees of freedom, also obtaining a significant value of 1.4 from the χ^2^/df calculation. Likewise, the descriptive goodness-of-fit indices corroborate the adequacy of the factorial model, again providing robust results for the Comparative Fit Index (CFI), with a value of 0.94, and for the RMSEA, which is 0.034 within the confidence interval 90% CI [0.029–0.039].

### 3.2. Relationship Between Psychopathy and Sensation Seeking

Next, the proposed hypotheses were tested. The first hypothesis, which established the existence of a positive relationship between the psychological variables under study, was evaluated using Spearman’s non-parametric Rho statistic, yielding the results shown in Table 3. As can be seen, the interpersonal and behavioural factors of the LSRP correlate positively with both BSS subscales, obtaining in both cases a higher correlation coefficient with the DES subscale (interpersonal–DES rs = 0.307) (behavioural–DES rs = 0.330) (interpersonal–BEM rs = 0.149) (behavioural–BEM rs = 0.150). In contrast, the affective factor of the LSRP correlates negatively with the DES subscale (rs = −0.300), while the correlation with BEM is not significant (*p* > 0.05). Finally, significant correlations are also observed between the factors within each of the scales, but this is irrelevant given that their factorial structure has already been verified as a measurement instrument.

### 3.3. Psychopathy and Sensation Seeking by Age

On the other hand, to test the second hypothesis, referring to the relationship between psychological variables and the sociodemographic variable ‘age,’ the Mann–Whitney U statistic was used. However, as shown in Table 4, the results were not statistically significant, so no differences were found between the two age groups. Spearman’s Rho statistic was used, finding a negative and low correlation coefficient for the behavioural factor of the LSRP with the age variable (rs = −0.122, *p* < 0.05).

### 3.4. Psychopathy and Sensation Seeking by Gender

Finally, the relationship between each of the psychological variables and the sociodemographic variable ‘gender’ was analysed using the Mann–Whitney U statistic. Statistically significant differences (*p* < 0.05) were observed between the two sexes in the behavioural factor of the LSRP and in the BSS subscales, with effect sizes (η^2^) of 0.015, 0.030 and 0.043, respectively. On the other hand, it should be noted that higher scores were obtained in the male group in the three factors that proved to be significant (see Table 5).

## 4. Discussion

The defining behaviours of psychopathy at a subclinical level are characteristic of impulsive, irresponsible individuals who are morally disconnected and socially aversive (Blötner et al., 2021), with deficits in self-control (Pechorro et al., 2022), limited collective interests, conservation, and self-transcendence (De Holanda Coelho et al., 2021). Specifically, our research interest has focused on analysing the relationship between psychopathic traits and sensation seeking, as well as the differential profiles based on gender and age.

### 4.1. Interpretation of the Relationships Between Psychopathy and Sensation Seeking

Regarding the first objective, there appear to be positive relationships between both psychological variables, except for the third factor of psychopathy, which correlated negatively with disinhibition. In the factors related to the interpersonal and behavioural facets of psychopathy, moderate correlation coefficients were obtained for the disinhibition subscale, which is understood as the desire to participate in social activities without restrictions, which could lead to the transgression of norms (Pérez de Albéniz et al., 2019). The relationship between this subscale and the interpersonal factor of psychopathic traits can be explained by the characteristics of the latter, which include an egocentric view of the world that predisposes the psychopath to actively seek their own satisfaction without regard for the violation of the rights and freedoms of others, accompanied by a lack of inhibition in the expression of threats or physical violence (R. Rodríguez & González-Trijueque, 2014). Likewise, in the case of the relationship between the behavioural component of psychopathy and disinhibition, this could be due to the impulsivity that characterises this psychopathic factor, understood as the sensitivity to regulate oneself mainly by signals of reward and immediate gratification without considering the costs that this may entail for others or for oneself (Díaz et al., 2018). In the case of thrill seeking, positive correlations are also obtained with the interpersonal and behavioural components of psychopathy, although in this case the values are low; hence, no inferences can be drawn.

On the other hand, contrary to the hypothesis, the affective factor of psychopathy showed a negative (also moderate) correlation with disinhibition. According to Weidacker et al. (2017), this relationship makes sense when considering the characteristic traits of this factor relating to a lack of empathy and emotional bonds with others, coldness in and control of situations, and inadequate anger management, which translates into an instrumental emotion aimed at controlling and subjugating others. All of this could lead the psychopath to commit acts of manipulation or instrumental violence with a high level of planning and persistence (B. Pérez et al., 2016; Sánchez, 2019). This is why a low score in disinhibition and a high score in the affective component could be considered to explain the high levels of premeditation in most of their goal-directed actions. In short, these results are consistent with the studies reviewed (see Alamán & Pérez, 2021; Rolls et al., 2022; Silva, 2009), which postulated that subjects with high scores in subclinical psychopathy would engage in reckless and sensation-seeking behaviours, with a lack of planning and irresponsibility towards themselves and others (interpersonal and behavioural components). All of this is accompanied by low empathy, which corresponds to the affective component.

### 4.2. Age Differences in Psychopathic and Impulsive Traits

Regarding the second objective, which focused on analysing age-related differences in the variables described, statistically significant, albeit weak, results were only obtained in the behavioural component of psychopathy. These results are consistent with previous findings (see Babad et al., 2021; Bentacourt & Campos, 2015), since, as hypothesised, this correlation was negative, thus indicating that the younger the age, the greater the psychopathic traits related to the behavioural component and impulsivity. Although there are discrepancies regarding the non-consideration of psychopathy until the age of majority due to its practical implications and life cycle events (Romero & Alba, 2019), studies (see Bentacourt & Campos, 2015; López-Romero et al., 2011) have been conducted that highlight the relationship between antisocial behaviour and adolescence, and between these factors and impulsivity and sensation seeking (see Babad et al., 2021). Based on these findings, it has been suggested that these behaviours are due to the implications of the maturational development at this stage and that these fundamentally neurobiological changes continue until the brain reaches maturity, which, although previously believed to occur around the age of 25, now extends to as late as 40 years of age (Somerville, 2016). For this reason, psychopathic and impulsive behaviours that are more prominent in young people tend to diminish as the brain matures over the years. Thus, behaviours associated with risk-taking and irresponsible and impulsive behaviours related to the need for stimulation and the search for immediate gratification decrease (Veloso, 2021). In turn, according to Stathi et al. (2021), recent research focuses on the subclinical area of psychopathic traits as predictors of negative social attitudes.

### 4.3. Inter-Gender Differences in Psychopathic and Impulsive Traits

With regard to the third and final objective, which studied the relationship between psychological variables and inter-gender differences, significant results were again obtained for psychopathy factors exclusively in the behavioural factor, with the presence of psychopathic traits being higher in men. These data confirm what has been reported in the scientific literature (see Anderson et al., 2021; Colins et al., 2017), which established a higher prevalence of psychopathic traits in men than in women, especially in reference to the behavioural and impulsive component. This result is consistent with Batool and Akhtar’s (2025) finding in university students, wherein men scored higher on psychopathy and sensation seeking. Although the symptoms and characteristics of subclinical psychopathy are similar in both genders, different behavioural manifestations are observed. While women are defined as more prone to the manipulation, egocentricity, and dishonesty characteristic of the affective and interpersonal component, men are better identified, as demonstrated in this research, with the impulsive component (see Barbosa-Torres et al., 2022; Suay, 2025). Specifically, they are associated with greater impulsivity, aggressiveness, behavioural problems and violent behaviours, as well as with the motivation for sensation seeking and sexual activity, presenting difficulties in impulse control (Reglero, 2020).

It should be noted that a statistically significant relationship was also found in the BEM and DES subscales of the Sensation Seeking Scale based on gender, with psychopathic traits being more prevalent in men in both cases. Once again, these results are consistent with previous research (see Bentacourt & Campos, 2015; Gil-Olarte et al., 2017; Palacios, 2015; Teva & Bermúdez, 2008), which established such differences in sensation-seeking behaviours. In these studies, as in those referring to age differences, biological and socialisation factors are cited to explain these results. These findings are consistent with previous ones, as men are characterised by impulsive, uninhibited behaviours motivated by the need for stimulation and immediate gratification, which could lead them to engage in risky activities to experience novel sensations and escape from routine (Batool & Akhtar, 2025; R. Rodríguez & González-Trijueque, 2014).

### 4.4. Limitations and Future Directions

This study is not without limitations. First, it should be noted that the study begins with the removal of two subscales in the case of the Sensation Seeking Scale (BSS). Furthermore, in the case of both the LSRP, which could be used in its entirety (with the exception of some items), and the subscales used from the BSS, the reliability coefficients were low. As specified in the ‘Measures’ Section, following an exploratory factor analysis, low reliability was observed for the emotion seeking (BEX) and susceptibility to boredom (SAB) subscales (with values of 0.48 and 0.55, respectively) of the Sensation Seeking Scale (Form V) (J. Pérez & Torrubia, 1986); therefore, they were not used in the corresponding analyses to avoid any potential impact on the validity of the findings. A possible explanation for this could be the outdated language in which the items are formulated, according to Chico and Vázquez (1999), although this should be interpreted with caution. With regard to the design, a cross-sectional design was used, although with this type of study it is not possible to establish causal relationships between variables (M. Rodríguez & Mendivelso, 2018). Similarly, non-probabilistic sampling is characterised by its limitations in relation to the representativeness of the population, which sometimes results in data distortion. Possible sampling limitations must also be taken into account.

However, taking all of the above into consideration, a line of future research opens up in which new, updated instruments could be developed. This new avenue should take into account the proven existence of a three-factor structure for psychopathy, as well as its relationship (both negative and positive) with sensation-seeking behaviours, which could be considered a predictor of the pathology. Likewise, the incorporation of measures of symptom exaggeration or social desirability could be considered in order to avoid the appearance of these biases that are often found in psychological instruments (Sáenz, 2020). The novelty of this study is that it interestingly studies the research variables in a non-forensic sample and in the age range, following the criteria of the American Academy of Pediatrics (see Sawyer et al., 2018), from late adolescence at ages 18 to 24 to young adulthood at ages 25 to 40 (American Academy of Pediatrics, 2019). Much of the research in the specialised literature on the general population has been conducted on adults (see Colins et al., 2017; Joubert, 2022; Sanz-García et al., 2021), not on adolescents and young adults as in the present study. Likewise, the analysis of the differential profiles in both variables based on gender and age is of interest.

Finally, although the present study focused on the relationship between sensation seeking and psychopathy, it did not include additional social, emotional, or problem-behaviour-related variables (e.g., socioeconomic status, emotion regulation, anxiety, empathy), which could further inform the understanding of these constructs. The absence of these variables represents a limitation of the current study and should be addressed in future research to provide a more comprehensive perspective.

## 5. Conclusions

This study confirmed the existence of a positive and significant relationship between the interpersonal, behavioural, and affective factors of psychopathy, measured using the Levenson Self-Report Psychopathy Scale (LSRP), and the ‘disinhibition’ subscale of the Sensation Seeking Scale (BSS). This means that behaviours oriented towards the desire to engage in social and risky activities can be found in individuals belonging to non-forensic populations with interpersonal and behavioural psychopathic traits, while in individuals with affective traits, these sensation-seeking behaviours will be reduced.

In contrast, no conclusive relationships were found between psychopathy factors and the ‘thrill seeking’ subscale, nor was it possible to determine the relationship between age and the manifestation of psychopathic and sensation-seeking behaviours.

Finally, the existence of inter-gender differences in the behavioural factor of psychopathy and in the ‘disinhibition’ and ‘thrill seeking’ subscales was confirmed. Specifically, it was found that men are characterised by more impulsive and aggressive behaviours, as well as a greater need for stimulation and immediate gratification.

With regard to potential interventions to improve the well-being of people with high subclinical psychopathic traits, authors such as Lobbestael et al. (2022) suggest that enhancing support and interest in others is a promising intervention. Given the complexity of psychopathy, the advisability of implementing preventive measures at the individual, psychosocial and community levels is emphasised (see Vitale, 2022).

## Figures and Tables

**Table 1:** Model goodness-of-fit indices on the Levenson Self-Report Psychopathy Scale (LSRP).

Model	χ^2^	df	*p*	CFI	RMSEA
LSRP	337.260	186	<0.001	0.919	0.048

**Table 2:** Goodness-of-fit indices for the Sensation Seeking Scale.

Model	χ^2^	df	*p*	CFI	RMSEA
BSS	1031.467	734	<0.001	0.945	0.034

**Table 3:** Correlational analysis between psychopathy and sensation-seeking factors.

Sperman’s Rho	Interpersonal	Behavioural	Affective	BEM	DES
Interpersonal	--				
Behavioural	0.449 **	--			
Affective	−0.336 **	−0.337 **	--		
BEM	0.149 **	0.150 **	−0.105	--	
DES	0.307 **	0.330 **	−0.300 **	0.408 **	--

** *p* < 0.001.

**Table 4:** Hypothesis testing for psychopathy and sensation seeking as a function of age.

Factor	Average		Average Range		Mann–Whitney U	*p*	Effect Size
	≤24	>24	≤24	>24			
Interpersonal	12.60	12.20	177.60	165.84	13,209.00	0.283	0.0033
Behavioural	16.12	15.54	178.45	164.53	13,031.00	0.204	0.0046
Affective	22.26	22.56	170.35	177.13	14,732.50	0.536	0.0011
BEM	5.50	5.28	176.28	167.90	13,486.00	0.444	0.0017
DES	4.43	4.36	174.68	170.39	13,823.00	0.695	0.0044

Note: Effect size corresponding to Cohen’s η^2^ (Cohen, 1992).

**Table 5:** Hypothesis testing for psychopathy and sensation seeking as a function of gender.

Factor	Average		Average Range		Mann–Whitney U	*p*	Effect Size
	Men	Women	Men	Women			
Interpersonal	12.91	12.13	185.68	164.55	16,032.50	0.053	0.0108
Behavioural	16.47	15.52	187.69	163.21	16,310.50	0.025 *	0.0145
Affective	21.92	22.68	165.93	177.71	13,308.00	0.280	0.0033
BEM	6.03	5.00	194.14	158.91	17,200.00	0.001 *	0.0302
DES	5.01	4.00	198.13	156.24	17,751.50	0.000 *	0.0430

Note: Effect size corresponding to Cohen’s η^2^ * *p* < 0.05.

## Data Availability

The data are contained within this article, and the recordings and raw datasets supporting the conclusions of this study will be made available by the corresponding author on request.
